# ERK8 is a novel HuR kinase that regulates tumour suppressor PDCD4 through a miR-21 dependent mechanism

**DOI:** 10.18632/oncotarget.6363

**Published:** 2015-11-22

**Authors:** Urszula Liwak-Muir, Christine C. Dobson, Thet Naing, Quinlan Wylie, Lucia Chehade, Stephen D. Baird, Pranesh K. Chakraborty, Martin Holcik

**Affiliations:** ^1^ Molecular Biomedicine Program, Children's Hospital of Eastern Ontario Research Institute, Ottawa, ON, Canada; ^2^ Department of Pediatrics, University of Ottawa, Ottawa, ON, Canada; ^3^ Newborn Screening Ontario, Children's Hospital of Eastern Ontario, University of Ottawa, Ottawa, ON, Canada

**Keywords:** tumour supressor, miRNA, kinase

## Abstract

Programmed cell death 4 (PDCD4) is a tumour suppressor implicated in cancer development and progression and was recently identified as a repressor of cap-independent translation of specific genes involved in the regulation of apoptosis. We show that the RNA-binding protein HuR binds to the PDCD4 3′UTR to protect it from miR-21-induced silencing. However, following H_2_O_2_ treatment, PDCD4 mRNA is degraded via miR-21 binding. Importantly, we identify HuR as a novel substrate of the ERK8 kinase pathway in response to H_2_O_2_ treatment. We show that phosphorylation of HuR by ERK8 prevents it from binding to PDCD4 mRNA and allows miR-21-mediated degradation of PDCD4.

## INTRODUCTION

RNA binding proteins (RBPs) have many important roles in the post-transcriptional control of RNAs including splicing, stabilization, translation and localization of multiple mRNA targets. Normally located in the nucleus, RBPs can accumulate in the cytoplasm in response to cellular stress to regulate specific mRNA targets, allowing the cell to recover from stress or to undergo apoptosis. HuR is a ubiquitously expressed RBP belonging to the Hu/embryonic lethal abnormal vision (ELAV) protein family [1]. HuR localizes primarily to the nucleus where it is involved in regulating mRNA splicing [2], export [3], and polyadenylation [4] *via* its three RNA recognition motifs (RRMs). Notably, a hinge region between RRM2 and RRM3 contains a nucleocytoplasmic shuttling domain that shuttles HuR into the cytoplasm in response to cellular stressors such as UV, arsenite, and hydrogen peroxide (H_2_O_2_) [5, 6]. The cytoplasmic accumulation of HuR allows it to modulate mRNA stability and translation [7-9]. HuR mainly functions by binding to AU-rich elements (AREs) in the 3′ untranslated regions (UTRs) of target mRNAs. However, HuR can also bind the 5′UTR, where it has been shown to either positively or negatively regulate translation. For example, HuR binds to the 5′UTR of IGF-IR and Bcl-xL to repress their translation [9, 10]. In contrast, binding of HuR enhances the IRES-mediated translation of XIAP [8]. In addition, HuR has been implicated in translational regulation through its ability to impact microRNAs, although the precise mechanism is not clear. In a competitive role, the binding of HuR to the mRNA may prevent miR/RISC (RNA-induced silencing complex) binding, thus resulting in stabilization of the target mRNA and an increase in translation [11]. Conversely, HuR binding may result in conformational changes in the mRNA that promote miR/RISC binding, leading to mRNA degradation or translation inhibition [11]. Given the diverse functions of HuR, it is no surprise that it plays a major role in the initiation and progression of cancer. This occurs mainly through its ability to regulate the stability or translation of target mRNAs involved in tumour growth, angiogenesis, invasion, and metastasis [12].

Programmed cell death 4 (PDCD4) is a tumour suppressor protein whose expression is increased during apoptosis [13], and has been implicated in the development of lung, colon, liver, breast, and brain cancers [14-18]. PDCD4 binds to and inhibits the eukaryotic initiation factor (eIF) 4A, the main helicase required for cap-dependent translation, suggesting a role as a general inhibitor of translation [19, 20]. In addition, PDCD4 was shown to inhibit the translation of several specific mRNA targets such as p53 [21], XIAP and Bcl-xL [22] through a cap-independent mechanism. We recently demonstrated that the loss of PDCD4 in Glioblastoma multiforme (GBM) tumours correlates with an increase in Bcl-xL expression, and that re-expression of PDCD4 results in down-regulated Bcl-xL expression and increased sensitivity to chemotherapeutics [18]. Determining the mechanism of PDCD4 regulation is crucial to better understand tumorigenesis. At the protein level, PDCD4 can be phosphorylated by S6 kinase 1 (S6K1) in response to mitogens [23] or S6K2 in response to fibroblast growth factor -2 (FGF-2) [22, 24], leading to its degradation. PDCD4 is also regulated at the mRNA level by microRNA (miR)-21, which is overexpressed in a variety of cancers [25-27].

Here, we describe a novel observation where HuR controls PDCD4 expression by regulating miR-21 binding to PDCD4 mRNA. We show that reducing HuR levels by siRNA results in a loss of PDCD4 that is mediated through miR-21. We further demonstrate that treatment of cells with H_2_O_2_ leads to the loss of PDCD4 that is executed through miR-21. We show that treatment of cells with H_2_O_2_ results in activation of Extracellular Signal Regulated Kinase 8 (ERK8, Mitogen-Activated Protein Kinase 15, MAPK15) and subsequent phosphorylation of HuR by ERK-8. Once phosphorylated, HuR loses its ability to bind the PDCD4 mRNA, thus making it available for miR-21-mediated repression.

## RESULTS

### HuR controls PDCD4 protein expression by regulating mRNA stability

**Figure 1:**
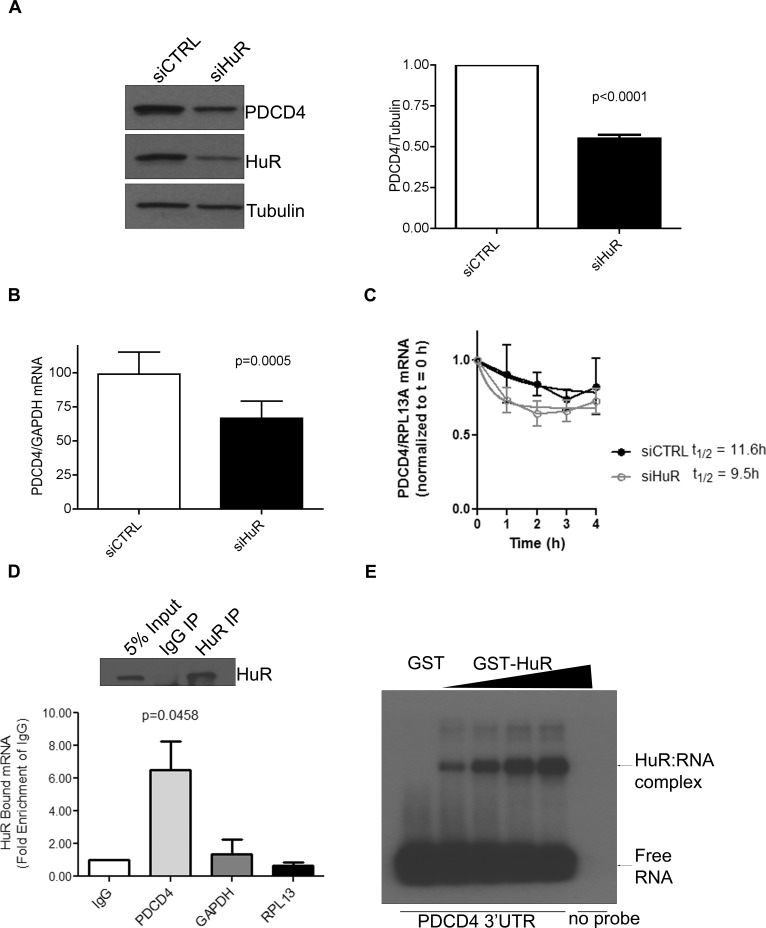
HuR directly binds to PDCD4 3′UTR mRNA to regulate its protein expression **A**. Left panel: Western blot analysis of PDCD4 protein levels after HuR knockdown. HeLa cells were treated with siHuR or siCTRL (non-targeting control) for 72 h and harvested for western blot analysis. Tubulin was used as a loading control. Right panel: PDCD4 protein levels are quantified relative to Tubulin. **B**. HeLa cells were treated with siHuR or siCTRL for 72 h, harvested, and total RNA was isolated. PDCD4 mRNA levels were quantified by qRT-PCR and are shown relative to GAPDH mRNA levels. **C**. Seventy-two hours after siRNA transfection, HeLa cells were treated with 5 μg/mL actinomycin D. After the chase period, cells were processed for qRT-PCR to determine the mRNA half-life (11.6h for siCTRL; 9.5h for siHuR). **D**. Top panel: HeLa cells were crosslinked with formaldehyde and endogenous HuR was immunoprecipitated with mouse anti-HuR antibody; IgG was used as a control. Western blot analysis shows the level of immunoprecipitated HuR. Bottom panel: HuR-bound RNA was isolated and quantified by qRT-PCR, and is shown relative to IgG-immunoprecipitated material. The levels of GAPDH and RPL13 in HuR immunoprecipitation were determined as specificity controls **E**. PDCD4 3′UTR RNA was *in vitro* transcribed, ^32^P labelled and UV crosslinking was performed with recombinant GST (control) or GST-HuR, separated by SDS-PAGE, and exposed to X-Ray film.

To better understand the role of HuR in regulating PDCD4, we transiently transfected HeLa cells with small interfering (si) RNA against HuR and observed a marked reduction in PDCD4 protein levels (Figure 1A). Since HuR is known to bind to AU-rich elements (ARE) in the 3′UTR regions of many mRNAs, and the 3′ UTR of PDCD4 is AU-rich (http://utrdb.ba.itb.cnr.it/) we measured the steady-state mRNA levels of PDCD4 after HuR knockdown. Indeed, we observed a ∼50% decrease in PDCD4 mRNA (Figure 1B) as compared to control. Additionally, we performed Actinomycin D experiments after HuR knockdown and calculated the half-life of PDCD4 mRNA as 11.6h in control cells and 9.5h after HuR knockdown (Figure 1C) which suggests that a loss of HuR results in the loss of PDCD4 mRNA stability. In order to identify if PDCD4 is a target mRNA that HuR specifically binds to, we immunoprecipitated endogenous HuR from HeLa cells and analyzed bound RNAs by qRT-PCR. We were able to successfully immunoprecipitate HuR (Figure 1D top panel) and isolation of bound RNAs followed by qRT-PCR identified that PDCD4 mRNA is enriched in HuR IP as compared to IgG control (Figure 1D bottom panel). It is possible that this observed interaction is indirect; therefore, we performed *in vitro* binding experiments with purified recombinant GST-tagged HuR and *in vitro* transcribed ^32^P-labelled PDCD4 3′UTR to determine if HuR can directly bind to the PDCD4 UTR and observed dose-dependent binding of HuR to the PDCD4 3′UTR (Figure 1E). These observations demonstrate that HuR regulates PDCD4 mRNA stability by directly binding to its 3′UTR.

### HuR regulates PDCD4 mRNA stability via miR-21

**Figure 2:**
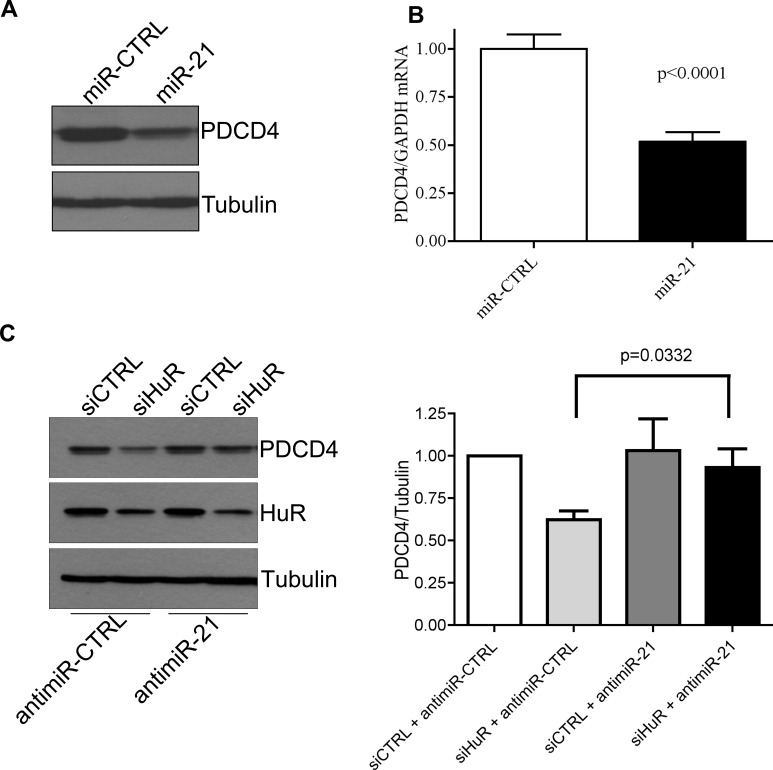
HuR regulates PDCD4 stability via miR-21 **A**. HeLa cells were transiently transfected with a miR-21 mimic for 24 h and cells were harvested for western blot analysis. Tubulin was used as a loading control. **B**. HeLa cells were transiently transfected with a miR-21 mimic for 24 h and RNA was harvested. qRT-PCR analysis showing decrease of PDCD4 mRNA relative to GAPDH after miR-21 over-expression. **C**. Left panel: AntimiR-21 or antimiR-CTRL (control) was transiently transfected into HeLa cells for 24 h followed by siHuR transfection for an additional 48 h. Cells were harvested and protein levels were analyzed by western blot. Right panel: Quantification of PDCD4 protein levels relative to Tubulin.

**Figure 3:**
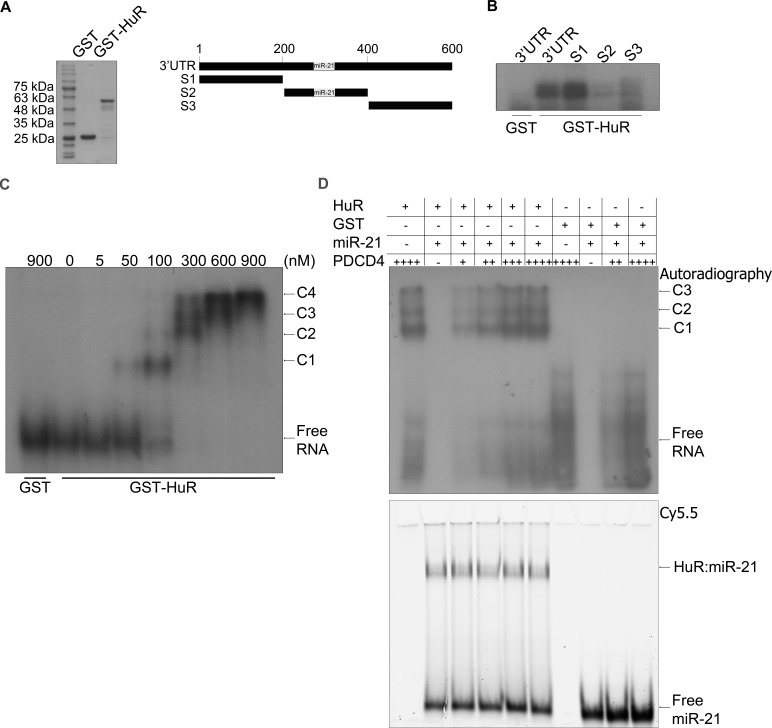
HuR oligomerizes on the PDCD4 3′UTR **A**. Left panel: Coomassie stain of the recombinant GST and GST-HuR purified from E. coli cells. Right panel: Schematic representation of a fragment of the PDCD4 3′UTR (nucleotides 1-610). S1: nucleotides 1-199, S2: nucleotides 200-400, S3: nucleotides 401-610. The grey box indicates the miR-21 binding site at nucleotides 228-249 [25]. **B**. UV-crosslinking with GST or GST-HuR and the PDCD4 3′UTR fragments that were *in vitro* transcribed and ^32^P-labelled. **C**. RNA EMSA with increasing concentrations of GST-HuR and *in vitro* transcribed and ^32^P-labelled PDCD4 S1S2 probe (nucleotides 1-401). The complexes between HuR and PDCD4 S1S2 RNA are indicated as C1, C2, C3, and C4. **D**. RNA EMSA with 300 nM GST-HuR or GST incubated with 8 nM Cy5.5 3′-end labelled miR-21 RNA and increasing concentrations of 5 pM, 10 pM, 15 pM, or 20 pM ^32^P-UTP labelled, *in vitro* transcribed S1S2 fragment of PDCD4 RNA. The complexes between HuR and PDCD4 S1S2 RNA are indicated by C1, C2, and C3. The binding between HuR and miR-21 is indicated by HuR:miR-21. Gel was exposed to X-ray film at −80°C to detect autoradiography and subsequently scanned with the Li-Cor Odyssey infrared scanner to detect the miR-21 Cy5.5 signal.

Recently, HuR has been implicated in regulating some mRNAs through their miR binding sites [11]. Since PDCD4 is a known target of miR-21 [25], we investigated the potential of HuR to regulate PDCD4 through miR-21. First, we confirmed that PDCD4 is a target of miR-21 by transiently transfecting a miR-21 mimic and observing a reduction in PDCD4 protein (Figure 2A) and mRNA (Figure 2B) levels. To determine if the effect of HuR knockdown on PDCD4 expression is mediated through miR-21, we overexpressed an anti-miR-21 (that binds to endogenous miR-21) to inhibit its activity. HuR knockdown in combination with a non-targeting antimiR-control showed a decrease in PDCD4 expression. In contrast, this reduction was blocked when cells were treated with the antimiR-21 (Figure 2C). This data suggests that HuR prevents miR-21 from binding to the PDCD4 mRNA resulting in protection of the mRNA from degradation. One possible mechanism is that HuR binds directly to the miR site on the 3′UTR thus blocking the miR from binding directly [11, 28]. To determine if HuR binds the miR-21 site on PDCD4 we generated three ∼200 nt fragments from the first 610 nucleotides of the PDCD4 3′UTR (Figure 3A right panel). Fragment S2 contains the miR-21 site highlighted in grey. We performed UV cross-linking experiments with purified GST-tagged HuR (Figure 3A left panel) and ^32^P-labelled *in vitro* transcribed RNA probes. Interestingly, HuR did not bind to the miR-21 containing fragment S2 (Figure 3B). Instead, HuR bound specifically to the first 200 nt S1 fragment. Moreover, HuR did not bind to the S3 fragment, which further supports the specificity of the HuR-PDCD4 mRNA interaction. Since HuR does not seem to interact with the miR-21 site, we were interested in determining if binding to the S1 fragment could cause multimerization of HuR on the mRNA to possibly inhibit the binding of the RISC complex. We performed RNA electromobility shift assays (EMSA) with increasing concentrations of purified GST-HuR and ^32^P-labelled S1-S2 fragment (Nucleotides 1-400; Figure 3C). We observed the formation of four complexes with increasing concentrations of GST-HuR suggesting that HuR binds to the first 200 nt of the PDCD4 3′UTR, and further multimerizes on the RNA. To further investigate the possible interplay between miR-21, HuR and PCDC4 mRNA, we used differentially labelled RNAs (Cy5.5-miR-21; ^32^P-PDCD4 3′ UTR) in an RNA-EMSA. Interestingly, although HuR binds miR-21 (Figure 3D) as observed by the Cy5.5 signal (bottom), the presence of miR-21 does not impair HuR's ability to bind to and oligomerize on the PDCD4 RNA as observed by autoradiography (top). Although the binding of miR-21 to PDCD4 3′ UTR has been reported previously [25], we were unable to detect binding of miR-21 to PDCD4 RNA due to the low sensitivity of the Cy5.5 label (data not shown).

### Loss of PDCD4 expression after H_2_O_2_ treatment is mediated through miR-21 and ERK8

**Figure 4:**
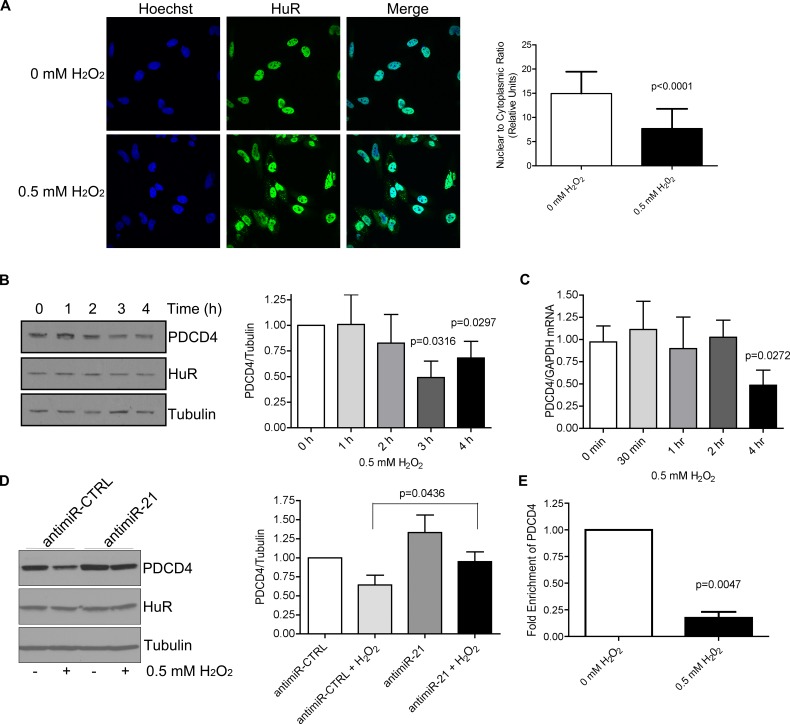
H_**2**_O_**2**_ causes cytoplasmic accumulation of HuR and a loss in PDCD4 expression that is mediated by miR-21 **A**. HuR localization by immunofluorescence of HeLa cells treated with PBS (0 mM H_2_O_2_) or 0.5 mM H_2_O_2_ for 1 h. Nuclei are visualized by Hoechst staining. Nuclear/Cytoplasmic ratio of HuR is shown on the right. Higher ratio denotes more nuclear staining. **B**. Left panel: HeLa cells were treated with 0.5 mM H_2_O_2_ for the indicated times and cell lysates analysed by western blot analysis indicating a decrease in PDCD4 protein at 3 h as compared to Tubulin control. Right panel: PDCD4 protein levels were quantified relative to Tubulin. **C**. Cells were treated with 0.5 mM H_2_O_2_ for the indicated time points, total RNA was isolated and analysed by qRT-PCR indicating a loss of PDCD4 mRNA as compared to GAPDH control. **D**. Left panel: HeLa cells were treated with antimiR-21 or a non-targeting antimiR-CTRL (control) for 24 h followed by treatment with 0.5 mM H_2_O_2_ for 4 h. Cells were harvested and analysed by western blot analysis. Tubulin was used as a loading control. Right panel: Quantification of PDCD4 levels relative to Tubulin. **E**. HeLa cells were treated with 0.5 mM H_2_O_2_ or PBS and HuR was immunoprecipitated. Bound RNA was isolated and qRT-PCR was performed to determine levels of PDCD4 mRNA. The levels of HuR-bound PDCD4 in PBS-treated cells were set as 1.

Many cellular stresses, such as oxidative stress, result in an accumulation of cytoplasmic HuR that is usually mediated by phosphorylation of HuR [11]. This cytoplasmic accumulation is necessary for HuR's ability to stabilize mRNAs or control translation by placing HuR in the same sub-cellular compartment as its target mRNA. Therefore, we were interested in determining the effect of increased cytoplasmic HuR levels on PDCD4 expression. We were expecting that an increase in cytoplasmic HuR would provide a protective effect against miR-21-dependent degradation, leading to increased PDCD4 expression. Contrary to our expectations, however, treatment of cells with H_2_O_2_ resulted in a loss in PDCD4 expression both at the protein (Figure 4B) and mRNA (Figure 4C) levels, even though HuR accumulated in the cytoplasm as monitored by immunofluorescence (Figure 4A). Additionally, we tested the requirement of miR-21 for PDCD4 loss during H_2_O_2_ treatment as it was shown previously that miR-21 levels increase following H_2_O_2_ treatment [29]. Cells were pretreated with antimiR-21 and then exposed to H_2_O_2_. Inhibition of miR-21 rescued PDCD4 protein expression after H_2_O_2_ treatment (Figure 4D), suggesting that miR-21 binding is responsible for the reduced PDCD4 expression in response to H_2_O_2_ stress. This result suggests that it is not necessarily the increase in cytoplasmic HuR that is important for PDCD4 regulation, but rather, modifications of the cytoplasmic HuR after H_2_O_2_ treatment that may affect its target binding. We therefore performed the HuR immunoprecipitation after H_2_O_2_ treatment and observed a loss of PDCD4 binding to HuR after H_2_O_2_ exposure (Figure 4E). These observations point toward a model where under normal growth conditions, a small amount of HuR that is normally found in the cytoplasm binds to the PDCD4 3′UTR and protects it from miR-21 mediated degradation. However, under oxidative stress, HuR is likely modified so that it can no longer bind to PDCD4 mRNA, thus allowing miR-21 to bind to the PDCD4 3′UTR leading to degradation of the mRNA and loss of protein expression.

**Figure 5:**
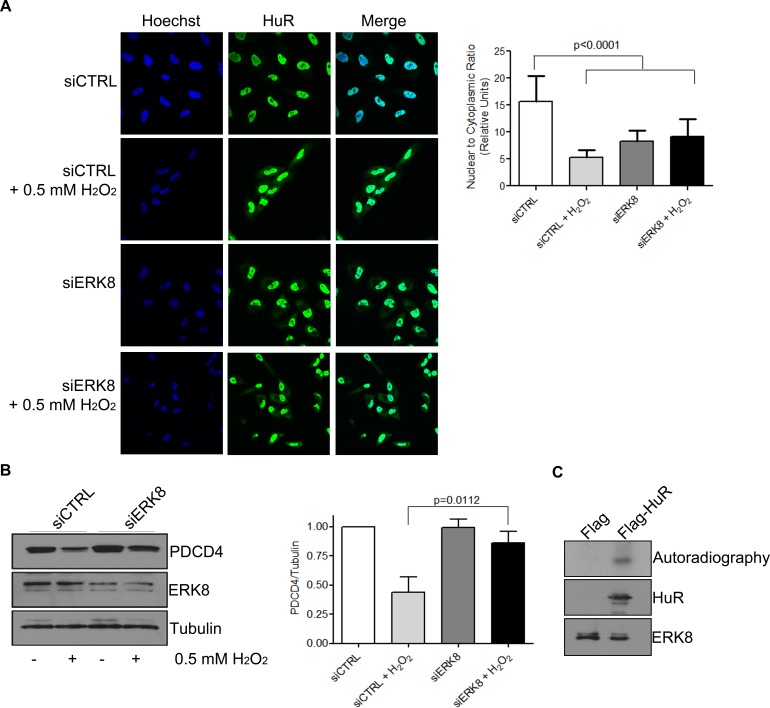
ERK8 phosphorylates HuR to prevent its binding to PDCD4 mRNA **A**. ERK8 or control siRNA was transfected into HeLa cells for 48 h followed by treatment of cells with 0.5 mM H_2_O_2_ or PBS for 1 h. Cells were fixed and immunofluorescence was performed to monitor HuR localization. Hoechst was used to stain the nuclei. Nuclear/Cytoplasmic ratio of HuR is shown on the right. Higher ratio denotes more nuclear staining. **B**. Top panel: HeLa cells were treated as in (A) and cells were harvested for western blot analysis for indicated proteins. Bottom panel: Quantification of PDCD4 protein levels relative to Tubulin. **C**. The kinase assay was performed with immunoprecipitated Flag-HuR or Flag empty vector as substrate and HA-ERK8 kinase in the presence of ^32^P gamma-ATP and exposed to X-ray film. The levels of HuR and ERK8 proteins were detected by western blot analysis.

Previous reports have identified the activation of the kinase ERK8 during H_2_O_2_ stress [30], thus we were interested in determining if HuR is a potential target of ERK8. We observed that PDCD4 protein levels are rescued after H_2_O_2_ treatment when ERK8 levels are reduced (Figure 5B), even though HuR still accumulates in the cytoplasm (Figure 5A). This data suggests that ERK8 may phosphorylate HuR to change its binding affinity for PDCD4. We therefore performed an *in vitro* kinase assay to determine if ERK8 can phosphorylate HuR directly. Indeed, we observed that HA-ERK8 specifically phosphorylates HuR, thus identifying HuR as a novel substrate of the ERK8 signaling pathway (Figure 5C). This data suggests a model where H_2_O_2_ causes activation of ERK8, which subsequently phosphorylates HuR, thus preventing it from binding to the PDCD4 3′UTR and rendering the PDCD4 mRNA accessible to miR-21 and leading to its degradation and loss of protein expression.

## DISCUSSION

Downregulation of the tumour suppressor PDCD4 is correlated with the initiation and progression of lung, colon, liver, breast, and brain cancers [14-18]. Previously, we identified PDCD4 as a regulator of IRES-mediated translation of XIAP and Bcl-xL [22]. This regulation is particularly important in cancer development because loss of PDCD4 correlates with an increase in the expression of these and other apoptosis-regulating proteins, thus contributing to the cell's ability to evade apoptosis following treatment with chemotherapeutics [18, 21]. The important role of PDCD4 in tumorigenesis highlights a need to elucidate the mechanism of PDCD4 protein regulation. It is known that miR-21 regulates PDCD4 mRNA and targets it for degradation, leading to a loss of protein expression [31]. Moreover, an increase in miR-21 expression has been observed in many cancers, which likely contributes to the frequently observed loss of PDCD4 [25-27]. Since HuR and PDCD4 regulate the same IRES-containing mRNAs [8, 9, 22], and HuR has been implicated in regulating miRNA-mediated degradation of mRNAs, we aimed to determine if HuR plays a role in regulating PDCD4 expression. Indeed, we observed that HuR regulates PDCD4 protein expression *via* miR-21. SiRNA-mediated loss of HuR renders PDCD4 mRNA readily available to miR-21 targeting, leading to its degradation and decreased protein levels. Interestingly, both the 3′ UTR of PDCD4 and the miR-21 target sequence are AU-rich, which is compatible with the HuR RNA target motif [32]. We demonstrate that HuR binds to the PDCD4 3′UTR but that it does not bind to the miR-21 binding site. Instead, our RNA EMSA analysis suggests that initial binding of HuR to the site upstream of the miR-21 binding site causes recruitment and further multimerization of additional HuR proteins that sterically hinder miR-21 binding. Furthermore, EMSA analysis suggests that HuR can bind both miR21 and PDCD4 mRNA. However, HuR has a higher affinity for the PDCD4 mRNA, which results in the observed effect on PDCD4 mRNA stability and protein expression.

After cellular stress, HuR exits the nucleus and accumulates in the cytoplasm where it typically binds to and stabilizes target mRNAs. Therefore, we were expecting to see a rescue in PDCD4 expression after exposing cells to H_2_O_2_ stress. Unexpectedly, although we observed a cytoplasmic accumulation of HuR following H_2_O_2_ treatment, we did not observe a rescue of PDCD4 expression. Instead, H_2_O_2_ treatment led to the loss in both PDCD4 protein and mRNA levels. This observation suggests that it is not the accumulation of HuR in the cytoplasm that is affecting PDCD4 levels, but rather, the modification of the already cytoplasmic HuR after H_2_O_2_ treatment that is causing the effect. It is possible that HuR is phosphorylated following H_2_O_2_, which prevents it from being able to bind to its target mRNA. A similar observation had been previously described by Abdelmohsen and colleagues [33], where they demonstrated that H_2_O_2_ causes phosphorylation of HuR by Chk2 thus leading to dissociation of HuR from SIRT1 mRNA and a consequent loss in SIRT1 protein. Additionally, Yoon and colleagues [34] demonstrated that tyrosine phosphorylation of HuR by JAK3 following arsenite treatment caused a dissociation of HuR from SIRT1 and VHL target mRNAs leading to their degradation. These findings point to a role for post-translational modifications of HuR in determining HuR's ability to bind target mRNAs. Since knockdown of Chk2 followed by H_2_O_2_ treatment had no effect of PDCD4 levels (data not shown) we sought to determine which kinase is regulating HuR's effect on PDCD4. Recently, ERK8 was shown to be activated in response to H_2_O_2_ stress [30]. Therefore, we monitored the effect of ERK8 silencing on PDCD4 levels. We determined that loss of ERK8 rescues the levels of PDCD4 protein after H_2_O_2_ treatment and that ERK8 specifically and directly phosphorylates HuR *in vitro*, thus identifying HuR as a novel target of ERK8 kinase activity.

Interestingly, although there is increased expression of both cytoplasmic HuR and miR-21 in primary Glioblastoma cells [27, 35-37], the expression of PDCD4 in these cells is reduced. While the ratio of cytoplasmic HuR and miR-21 will determine the fate of PDCD4 mRNA, we have shown that the binding affinity of HuR is also critical for PDCD4 regulation. Therefore, in addition to monitoring levels of HuR and miR-21, one should consider assessing the activity of ERK8 and/or other kinases that may phosphorylate HuR to alter its binding affinity for specific targets.

## MATERIALS AND METHODS

### Cell culture, expression constructs, and transfection

HeLa cells were maintained in standard conditions in Dulbecco's modified Eagle's medium (DMEM) supplemented with heat-inactivated 10% fetal calf serum, 2 mM L-glutamine, and 1% antibiotics (100 units/ml penicillin-streptomycin). The GST-HuR expression plasmid was described previously [38]. Transfections of siRNA, or miRVana microRNA mimics and inhibitors were performed using Lipofectamine RNAiMax (Life Technologies). Briefly, 2.5 × 10^4^ HeLa cells were seeded in 12-well plates for 24 hours. Transfections were performed at a final concentration of 20 nM HuR siRNA (AAGUCUGUUCAGCAGCAUUGGUUdTdT, Dharmacon), nonsilencing control (Qiagen, Cat. # 1022076), miR-21 mimic (Ambion, Cat. # 4464066), miR negative Control mimic (Ambion, Cat. # 4464058), anti-miR-21 (Ambion, Cat. # 4464084), anti-miR control (Ambion, Cat. # 4464076). Cells were treated in the presence of 0.5 mM H_2_O_2_ for 4 hours. Cells were harvested for analysis after the indicated time points as described below.

### Western blot analysis

Cells were washed with PBS, scraped, and transferred to an Eppendorf tube. Cells were pelleted and resuspended in RIPA buffer (50 mM Tris-HCl [pH 7.4], 1 mM EDTA, 150 mM NaCl, 1% NP-40, 0.5% SDS, 1 mM PMSF) for 15 minutes on ice. Lysates were centrifuged at 12,000 x g for 15 minutes to pellet cell debris. Bradford Assay (Bio-Rad) was used to quantify protein concentration and equal concentrations were loaded on 10% SDS-PAGE gels. Proteins were transferred to a PVDF membrane and analysed by rabbit anti-PDCD4 (Rockland, CAT# 600-401-965), mouse anti-HuR (Santa Cruz Biotechnology, CAT# sc-5261), mouse anti-Tubulin (Abcam, CAT# ab7291), rabbit anti-GST (Santa Cruz Biotechnology, sc-459), and goat anti-ERK8 (Santa Cruz Biotechnology, CAT# sc-86723) antibodies followed by species-specific HRP-conjugated secondary antibodies (Cell Signaling Technology). Antibody complexes were detected using an ECL or ECL Plus system (GE Biosciences) and were quantified using Odyssey densitometry software (Li-COR Biosciences).

### RNA extraction and quantitative RT-PCR (qRT-PCR) analysis

Total RNA was isolated from cells using RNAzol (Molecular Research Center, Inc.) as per manufacturer's protocol. cDNA was generated using the First-strand cDNA synthesis kit (GE Biosciences). Quantitative PCR was performed using the QuantiTect SYBR green PCR kit (Qiagen) with gene specific primers for PDCD4 (QuantiTect Primer Assay; Qiagen) and GAPDH [22].

### Actinomycin D

Seventy-two hours after siRNA transfection, HeLa cells were treated with actinomycin D (Sigma-Aldrich), dissolved in anhydrous ethanol, at a final concentration of 5 μg/mL. After the chase period, cells were processed for qRT-PCR to determine their half-life (*t*_1/2_) as described [39]

### *In vitro* synthesis of ^32^P-labelled RNA and UV-crosslinking

The first 610 nt of the PDCD4 3′UTR containing the miR-21 binding site (228-249 nt; [25]) was cloned after the chloramphenicol acetyl transferase (CAT) coding sequence in the pMC.pa plasmid described in [39] using the forward (5′-CAGGATCCATATAAGAACTCTTGCAGTC) and the reverse (5′-CTTCTAGAACCAGGTTCATTTTTCC) primers. DNA templates containing the T7 promoter were generated from this pMC.PDCD4_3′UTR.pa plasmid by PCR (S1 fragment: forward primer 5′-CAGGATCCATATAAGAACTCTTGCAGTC, reverse primer 5′-CTTCTAGACTTGCCCCCTCGAAAAAC; S2 fragment: forward primer 5′-CAGGATCCGAGGGACAGAAAAGTAAC, reverse primer 5′-CTTCTAGATTTTAGCAGCTTAACTTT; S3 fragment: forward primer 5′-CAGGATCCCCCCATGTTGGCTGCTGC, reverse primer 5′-GGAAAAATGAACCTGGTTCTAGAAG). RNA was generated using [α-^32^P]UTP and a MAXIscript T7 kit (Ambion) as per the manufacturer's protocol. The RNA was run on a 5% acrylamide–8 M urea denaturing gel, excised and eluted in RNase free water overnight at 37°C. The RNA was then incubated with purified GST or GST-HuR in RNA binding buffer (10 mM Tris-HCl [pH 7.4], 3 mM MgCl_2_, 300 mM KCl, 1 mM dithiothreitol [DTT], 0.2 mM PMSF, leupeptin [20 μg/ml]) for 30 minutes at room temperature then cross-linked at 250 mJ/μm^2^ in a Stratalinker. The complexes were treated with RNase T1 (1 U/μl), RNase A (10 μg/ml), and heparin (5 mg/ml) for 10 minutes. The samples were separated by SDS-PAGE gel, and exposed to X-ray film at −80°C overnight.

### GST-tag protein purification

E. coli was transformed with the pGEX or pGEX-KG_HuR plasmid and grown overnight in 4 mL Luria-Bertani (LB) media containing ampicillin (100 mg/mL). The culture was added to 100 mL of LB media containing 100 mg/mL of ampicillin and grown to an OD of 0.7. Isopropylthio-β-galactoside (IPTG) was added to a final concentration of 1 mM and grown for 4 hours longer. The cultures were centrifuged at 5000 x g for 10 minutes at 4°C and supernatant discarded. The pellet was resuspended in 10 mL of ice-cold PBS and the samples were centrifuged again at 5000 x g for 10 minutes at 4°C. Samples were then lysed with 10 mL lysis buffer (50 mM Tris-HCl [pH 8.0], 200 mM NaCl, 1 mM EDTA, 1 mM DTT, 2 mM PMSF). Lysates were sonicated twice, 1% Triton X-100 was added, and samples were sonicated again. Samples were centrifuged at 13000 x g for 10 minutes at 4°C. Glutathione sepharose beads (200 μL; GE Healthcare) were added to the supernatant and rotated at 4°C for 2 hours. Samples were washed 5 times with cold PBS and proteins were eluted using 20 mM L-glutathione, pH 8.0 in PBS rotating at 4°C for 1 hour.

### RNA-protein complex immunoprecipitation

HeLa cells were treated with 0.5 mM H_2_O_2_ for 4 hours, washed twice with PBS and lysed in CHIP lysis buffer (50 mM Hepes-KOH pH 7.5, 140 mM NaCl, 1 mM EDTA pH 8.0, 1% Triton X-100, 0.1% SDS, 0.1% sodium deoxycholate, 1 mM PMSF, 5 μg/mL Aprotinin, 10 μg/mL Leupeptin, 40 U/mL RNase inhibitor) for 30 minutes on ice. Lysates were spun at 13, 000 rpm for 15 minutes and supernatant was transferred to a new tube. Samples were incubated with 10 μg mouse anti-HuR or anti-mouse IgG for 2 hours at 4°C. Dynabeads Protein G (Novex by Life Technologies) were washed with CHIP buffer and added to samples (50 μL per sample) and rotated for 40 minutes at 4°C. Supernatant was removed and beads were washed 4 times with CHIP buffer. RNA was extracted with RNAzol as per manufacturer's protocol and qPCR was performed using the QuantiTect SYBR green PCR kit (Qiagen).

### Immunofluorescence

Cells were grown on coverslips and treated with H_2_O_2_ or PBS as indicated and fixed with 3% formaldehyde in PBS for 10 minutes at room temperature. Cells were permeablized with 0.1% Triton X-100 in PBS for 5 minutes at room temperature on a shaker, rinsed twice with PBS, and blocked with 5% bovine serum albumin (BSA) in PBS for 1 hour. Primary antibody was added (mouse anti-HuR (1:500 dilution in 5% BSA in PBS) and incubated with cells overnight at 4°C, followed by 3 washes with PBS for 5 minutes each. Secondary antibody (alexa fluor 488 goat anti-mouse (Life Technologies; 1:1000 dilution in PBS) was added for 1 hour followed by three 5 minute washes in PBS. Nuclei were stained with Hoechst 33342 (Pierce) for 5 minutes and washed with PBS twice. Coverslips were mounted on slides using Fluoromount (Sigma Aldrich). Confocal microscopy was performed using the 60X objective with immersion oil (Olympus Fluoview FV1000, Richmond Hill, Ontario Canada). Quantification of HuR nuclear and cytoplasmic distribution was done as described previously [40] with some modifications. The images were analyzed on a Columbus Image Analysis Server (Perkin Elmer) using an embedded Acapella Image Analysis Software (Perkin Elmer) script. Nuclei were segmented and defined using their Hoechst 33342 staining. The cytoplasmic region was defined as a ring of 9 pixel width that encircled the nucleus 1 pixels away from its outside edge. The nuclear/cytoplasmic ratio of HuR was calculated from the average intensity of HuR fluorescent signal measured per cell within these regions. Higher ratio number represent more nuclear distribution.

### RNA electromobility shift assay

Recombinant GST or GST-HuR was incubated with ^32^P-labelled, *in vitro* transcribed RNA probe in RNA binding buffer (10 mM Tris-HCl PH 7.5, 1.5 mM MgCl_2_, 50 mM KCl, 0.5 mM DTT) for 30 minutes at room temperature with or without Cy5.5 3′-labelled miR21 RNA (Dharmacon). The complexes were separated on a 6% polyacrylamide gel and the gel was exposed to X-ray film at −80°C to detect autoradiography and subsequently scanned with the Li-Cor Odyssey infrared scanner (Li-Cor Biosciences, Lincoln, NE) to detect the miR21 Cy5.5 signal.

### Kinase assay

pCDNA3_Flag-HuR, pReciever_HA-ERK8, or pCDNA3_Flag empty vector were transfected into HeLa cells for 24 h and harvested in co-immunoprecipitation buffer (25 mM Tris [pH 7.5], 150 mM NaCl, 50 mM NaF, 0.5 mM EDTA [pH 8.0], 0.5% Triton X-100, 5 mM beta glycerophosphate, 5% glycerol, 1 mM DTT, 1 mM PMSF, 1 mM Na_3_VO_4_). Lysates were sonicated and centrifuged for 15 min at 13,000 x g at 4°C. Anti-Flag agarose beads (Sigma) were incubated with the lysate for 1 h at 4°C and washed 3 times in lysis buffer followed by a wash in kinase buffer (20 mM Tris-HCl [pH 7.5], 5 mM beta-glycerolphosphate, 0.2 mM Na_3_VO_4_, 0.5 mM DTT). Kinase and substrate on beads were incubated in kinase buffer in the presence of (30 μM ATP, 6.6 mM MgCl_2_, 3.3 mM MnCl_2_) and 5 μCi of γ-^32^P-labelled ATP for 20 min at 30°C. Laemmli sample buffer was added, and samples were separated by SDS-PAGE, transferred to a PVDF membrane, and exposed to X-ray film. The membrane was subsequently analyzed by Western blotting.

### Statistical analysis

An unpaired t-test was performed using GraphPad Prism version 5.00 for Windows (GraphPad Software, San Diego, CA) to determine p-value in repeated experiments. All results are shown as mean ± standard deviation. For RT-qPCR experiments, average RNA expression was calculated using data collected from three biological replicates and three technical replicates for each biological replicate. Unless otherwise noted, all results were obtained through a minimum of three independent experimental replications.
